# Supplementary figures and images for: Med14 phosphorylation shapes genomic response to GLP-1 Agonist
Source: bioRxiv. 2025 Jun 23:2025.06.17.660196. Preprint. [Version 1] doi: 10.1101/2025.06.17.660196 (PMC12262311; doi:10.1101/2025.06.17.660196)

Figure S1

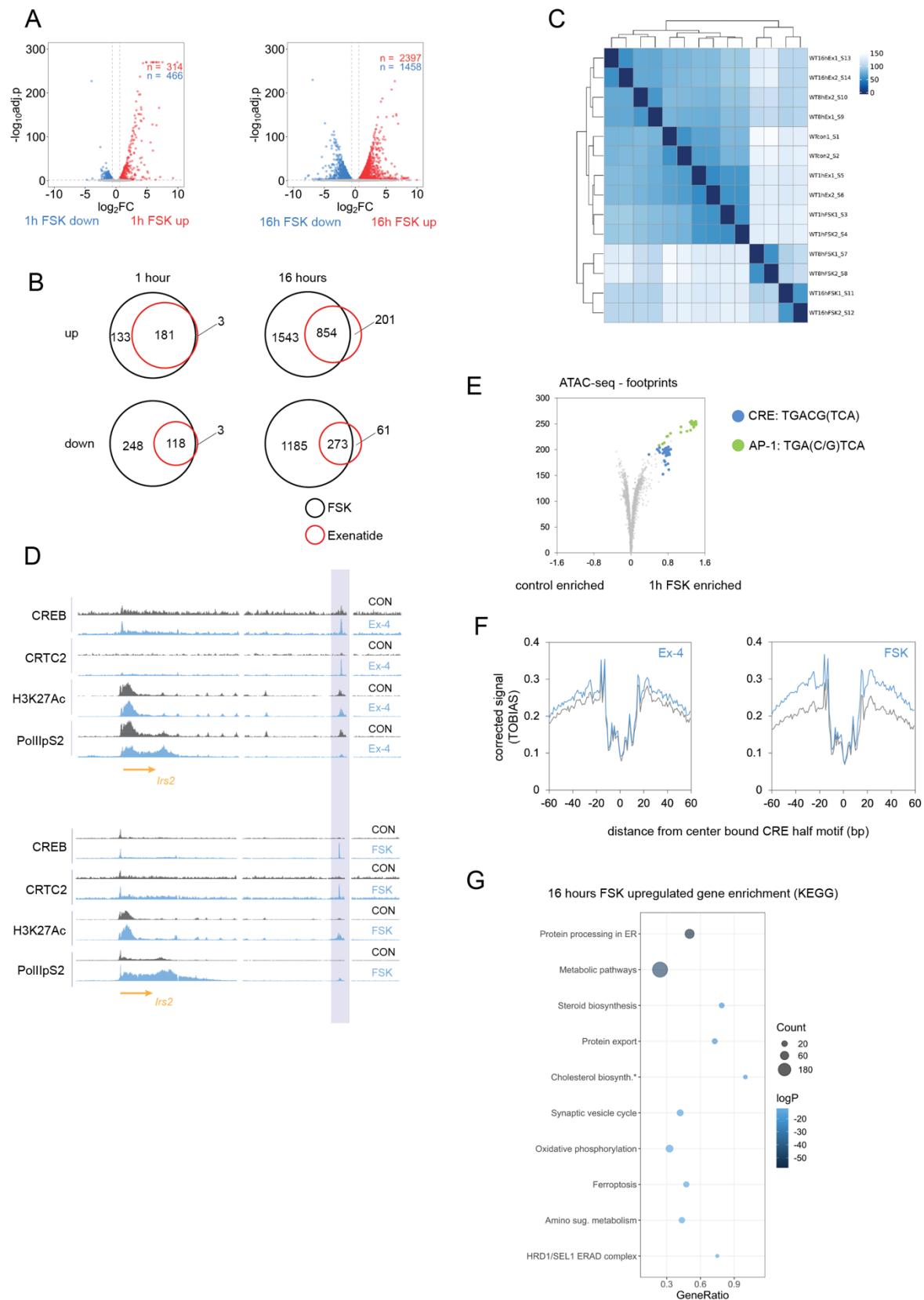

Figure S2

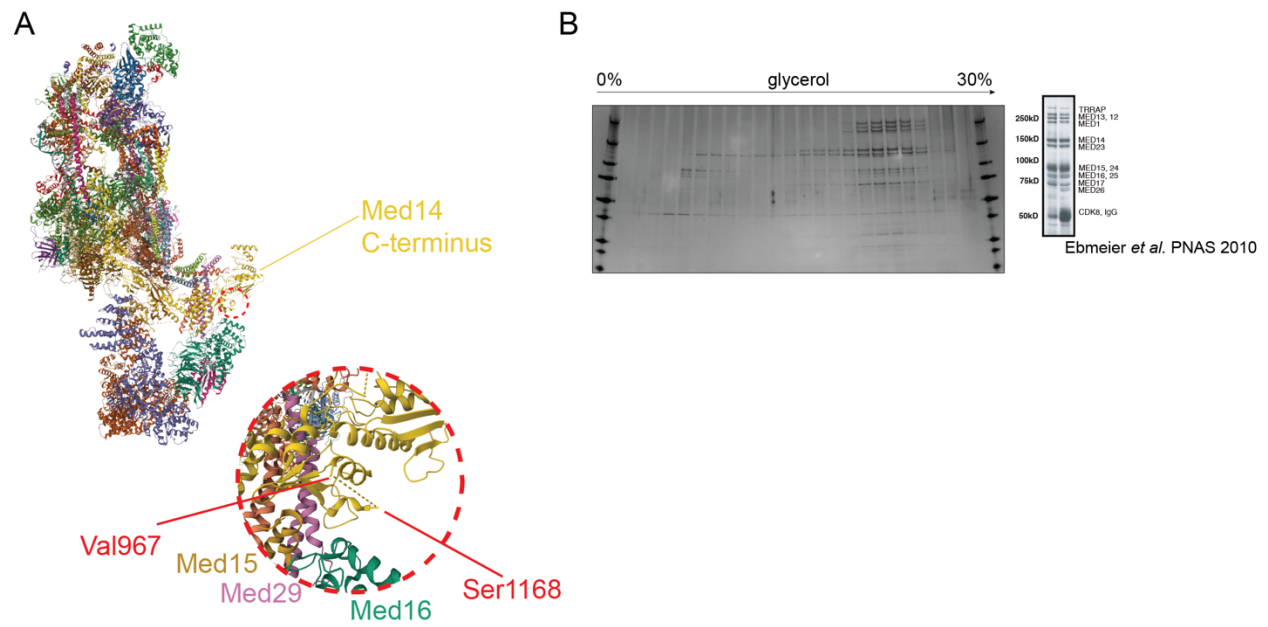

Figure S3

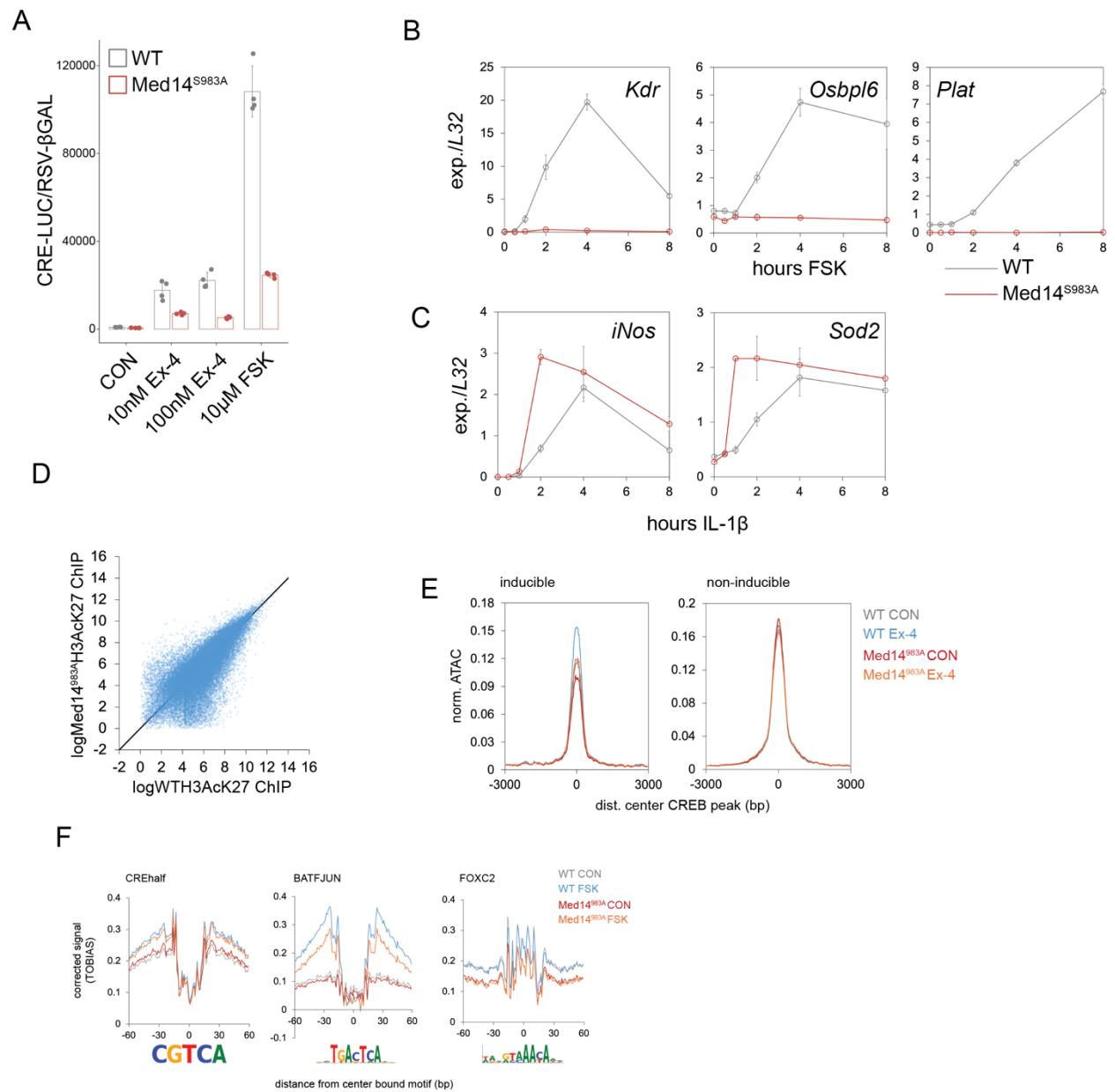

Figure S5

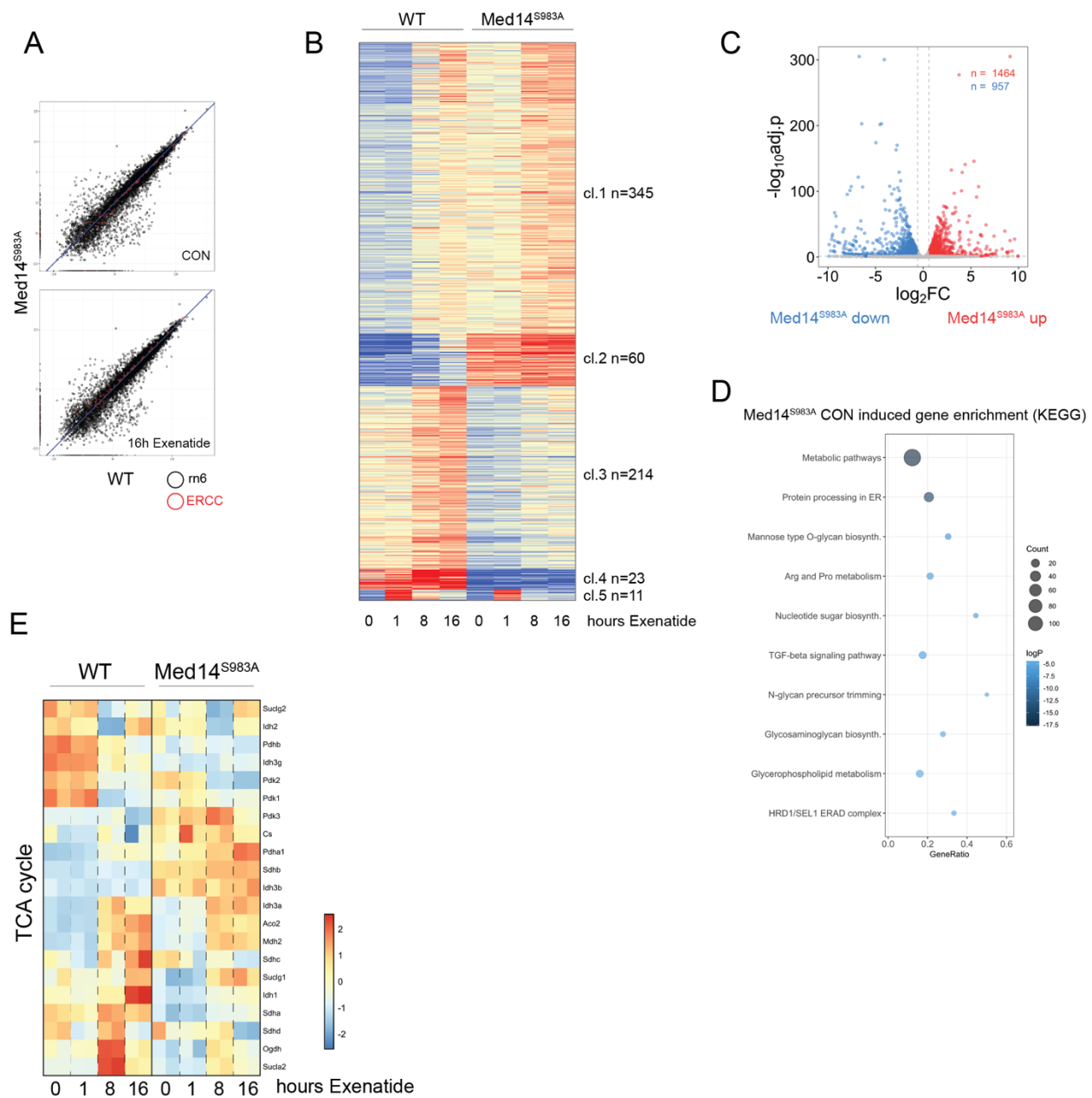

Figure S6

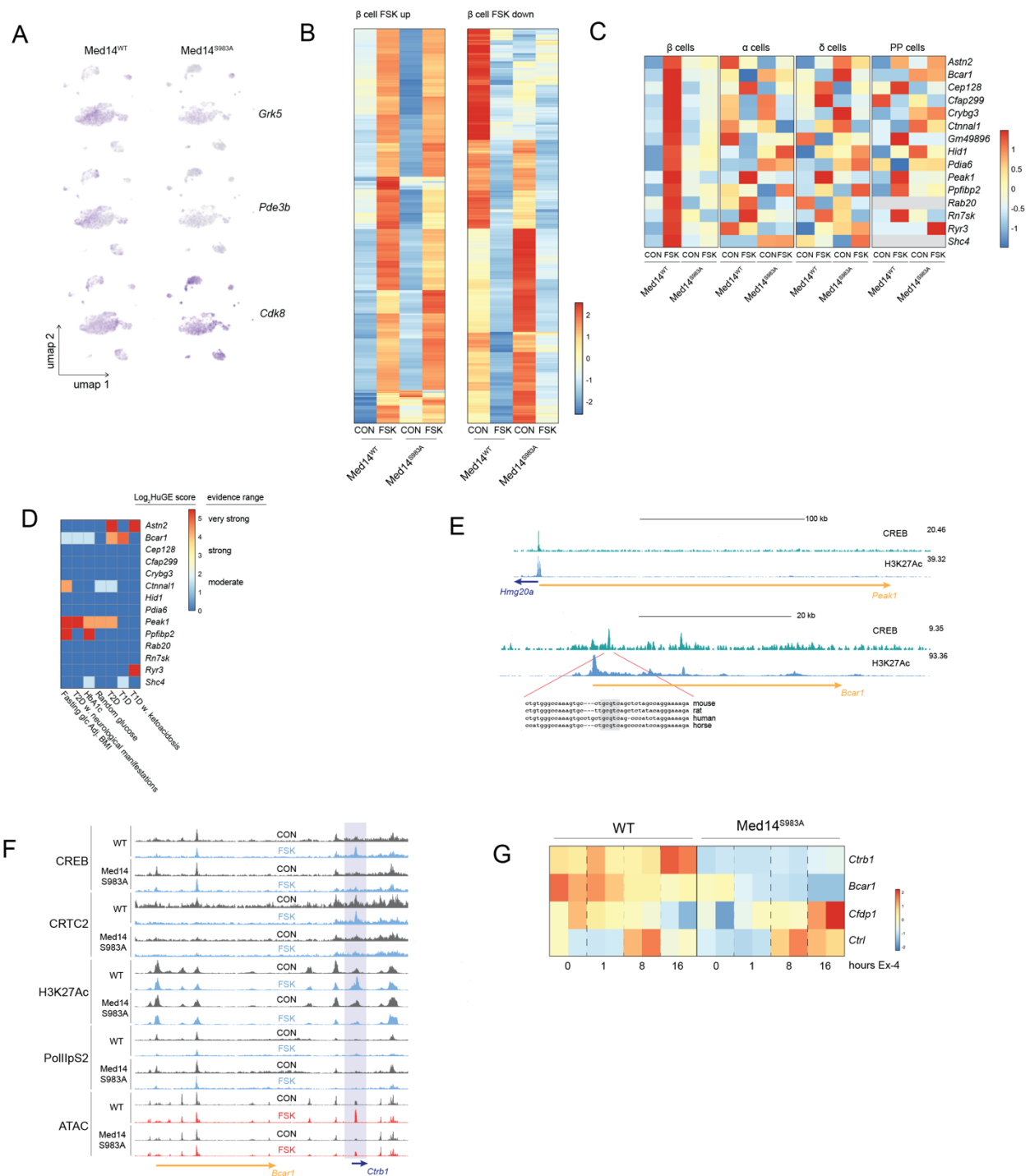

Supplement: 18 — Figure S1: Overlap between Ex-4 and forskolin (FSK) transcriptional responses. (A) Differential gene expression after acute (1h, left) and sustained (16h, right) FSK exposure in INS-1 cells. (B) Overlap of Ex-4 and FSK in induced (top) and repressed (bottom) genes after 1 hour (left) and 16 hour (right) exposure. (C) Sample similarity across all conditions. (D) Example ChIPseq tracks over the Irs2 locus showing binding of CREB and CRTC induced by Ex-4 (top) and FSK (bottom) over an activated distal enhancer (shaded). Enhancer activity and CTD-phosphorylated RNA polymerase II are shown in H3AcK27 and PolIIpS2 tracks, respectively. (E) Volcano plot depicting change in footprint score over transcription factor binding motifs in accessible chromatin regions (ATAC) after 1 hour FSK treatment. Motifs corresponding to CREB response binding (CRE) and activator protein-1 (AP-1) are highlighted. (F) ATACseq footprints over CRE half (CGTCA) sites after 1 hour Ex-4 (10 nM) (left) and FSK (10 μM) (right) treatment. (G)KEGG pathway enrichment analysis of genes induced after 16 hour FSK (10 μM) exposure. Figure S2: Med14 is a PKA target. (A) Structure of human mediator bound to pre-initiation complex (PDB 7LBM)[16]. Exposed Med14 CTD with unresolved IDR (Med14V967-S1168) is highlighted. (B) Silver stain of purified mediator complex with Med14 S983A loaded on a 10%-30% glycerol gradient. Mediator silver stain form Ebmeier et al. [80] shown for reference. Figure S3: Med14S983 phosphorylation promotes gene induction by activating enhancers. (A) Activation assay of WT and Med14 S983A cells transfected with a CREB-dependent (8xCRE) luciferase reporter. Cells were treated with Ex-4 or forskolin (FSK) as indicated for 6 hours. Signal was normalized by co-transfection of an RSV-βGAL construct. (B) Time course Q-PCR for delayed-early (Kdr, Osbpl6, Plat) genes over 8 hour FSK (10 μM) treatment in WT and Med14 S983A mutant cells. Error bars show standard deviation. (C) Time course Q-PCR for [file NIHPP2025.06.17.660196v1-supplement-18.pdf]
